# Vestibular schwannoma surgery

**DOI:** 10.3171/2021.7.FOCVID21148

**Published:** 2021-10-01

**Authors:** Michael J. Link, Isaac Yang, Fred G. Barker, Amir Samii, Philip V. Theodosopoulos

**Affiliations:** 1 Department of Neurosurgery, Mayo Clinic, Rochester, Minnesota;; 2 Department of Neurosurgery, Otolaryngology and Radiation Oncology, Ronald Reagan UCLA Medical Center, Los Angeles, California;; 3 Department of Neurosurgery, Massachusetts General Hospital, Boston, Massachusetts;; 4 Department of Neurosurgery, International Neuroscience Institute, Hannover, Germany; and; 5 Department of Neurological Surgery, University of California, San Francisco, California

## INTRODUCTION

Welcome to the October issue of *Neurosurgical Focus: Video*, regarding microsurgery for vestibular schwannomas (VSs). We are delighted by the enthusiastic response that the call for videos created. We received more than 50 video submissions from all over the world demonstrating various techniques and emphasizing many of the important nuances in these challenging operations. After a great deal of debate and deliberation, we selected the top videos that we believed would be of greatest interest to the viewership.

On the one hand, an operation to remove a VS always has the same straightforward goals: complete extirpation of the tumor and preservation of neurologic function. Yet, collectively, after doing thousands of these operations, the one thing that we agree on is that no two operations are ever the same. These videos demonstrate many of the unique aspects that the surgeon can encounter and ways to deal with challenging issues such as aberrant and adherent facial nerves. All three primary surgical approaches—middle fossa, retrosigmoid, and translabyrinthine surgeries—are highlighted. Videos dealing with VS and rare secondary effects such as hemifacial spasm and trigeminal neuralgia are included. Techniques to deal with cystic tumors, giant tumors, and aberrant anatomy such as a high jugular bulb, making the operation even more difficult, are reviewed. Surgical adjuvants such as the use of endoscopes are also highlighted. Finally, strategies to help deal with tumors related to neurofibromatosis type 2 are included, and hearing rehabilitation options with cochlear implants or auditory brainstem implants are discussed.

While some aspects of the operations remain constant, such as the release of CSF from the cisterns to get posterior fossa relaxation, the many nuances included in this issue’s videos can help us as surgeons to meet each case’s special challenges. We hope you enjoy this issue.

## Disclosures

Dr. Yang reports receiving grants from Stryker and Brainlab and personal fees from Baxter outside the submitted work.

